# Clozapine-Induced Chemogenetic Neuromodulation Rescues Post-Stroke Deficits After Chronic Capsular Infarct

**DOI:** 10.1007/s12975-022-01059-8

**Published:** 2022-07-09

**Authors:** Jongwook Cho, Seungjun Ryu, Sunwoo Lee, Junsoo Kim, Ji-Young Park, Hyuk-Sang Kwon, Hyoung-Ihl Kim

**Affiliations:** Department of Biomedical Science and Engineering, Gwangju Institute of Science and Technology (GIST), Gwangju, 61005 Republic of Korea

**Keywords:** Chronic stroke model, Chemogenetic neuromodulation, FDG-PET, Clozapine, Functional recovery

## Abstract

Long-term disabilities induced by stroke impose a heavy burden on patients, families, caregivers, and public health systems. Extensive studies have demonstrated the therapeutic value of neuromodulation in enhancing post-stroke recovery. Among them, chemogenetic neuromodulation activated by clozapine-N-oxide (CNO) has been proposed as the potential tool of neuromodulation. However, recent evidence showed that CNO does not cross the blood − brain barrier and may in fact have low binding affinity for chemogenetic tool. Thus, clozapine (CLZ) has been suggested for use in chemogenetic neuromodulation, in place of CNO, because it readily crosses the blood–brain barrier. Previously we reported that low doses of CLZ (0.1 mg/kg) successfully induced neural responses without off-target effects. Here, we show that low-dose clozapine (0.1 mg/kg) can induce prolonged chemogenetic activation while avoiding permeability issues and minimizing off-target effects. In addition, clozapine-induced excitatory chemogenetic neuromodulation (CLZ-ChemoNM) of sensory-parietal cortex with hsyn-hM3Dq-YFP-enhanced motor recovery in a chronic capsular infarct model of stroke in rats, improving post-stroke behavioral scores to 56% of pre-infarct levels. Longitudinal 2-deoxy-2-[18F]-fluoro-D-glucose microPET (FDG-microPET) scans showed that a reduction in diaschisis volume and activation of corticostriatal circuits were both correlated with post-stroke recovery. We also found c-Fos increases in bilateral cortices and BDNF increases in the cortices and striatum after CLZ-ChemoNM, indicating an increase in neural plasticity. These findings suggest the translational feasibility of CLZ-ChemoNM for augmenting recovery in chronic stroke.

**Supplementary Information:**

The online version contains supplementary material available at 10.1007/s12975-022-01059-8.

## Introduction

Stroke is a devastating clinical condition and the second leading cause of death worldwide [1]. As well as high mortality, strokes lead to prolonged disability among post-stroke survivors, imposing debilitating costs on their families, caregivers, and public healthcare systems [2, 3]. Despite substantial advances in the prevention and acute management of stroke, there are few options for attenuating prolonged neurological deficits; post-stroke neural plasticity imposes a limited “time window” for functional restoration and rehabilitation [4]. Many forms of brain stimulation have been explored to overcome the limits of conventional rehabilitation measures beyond this time window in chronic stroke patients, including repetitive transcranial magnetic stimulation (TMS), theta burst stimulation, epidural cortical stimulation, transcranial direct current stimulation (tDCS), transcranial alternating current stimulation (tACS), stimulation via a laser-based device, and vagal nerve stimulation [5, 6]. However, the results remain controversial [7–9].

Recently, chemogenetic and optogenetic neuromodulation have revolutionized brain stimulation because these tools allow selective modulation of neural activity and mapping of brain circuits. Cortical stimulation using these new techniques has significant potential to treat brain disorders that require induction of brain plasticity (e.g., stroke) [10–12]. Optogenetic stimulation provides on–off control of neurons or neural circuits with millisecond precision; however, this technique requires not only the implantation of optical devices targeting the particular brain area but also the delivery of a considerable level of energy during prolonged stimulation [13]. By contrast, clozapine-N-oxide (CNO)-activated chemogenetic modulation does not require an optical implant since it utilizes energy-conserving G-protein-coupled receptors (designer receptors exclusively activated by designer drugs, or DREADDs) [14]; rather, CNO is administered orally or injected through the intravenous or intraperitoneal routes. Furthermore, CNO-induced activation persists for several hours, which is critical for the induction of neural plasticity. Thus, chemogenetic neuromodulation is more readily translatable for the treatment of neurological disorders in humans.

Chemogenetic neuromodulation has been used extensively to study the brain circuits and mechanisms underlying feeding, sleep, anxiety, depression, and movement [14]. However, recent reports have questioned the mechanism of DREADD activation by CNO, arguing that CNO cannot directly cross the blood–brain barrier [15]. Such reports suggest that activation of DREADDs in vivo is likely mediated by the conversion of CNO to clozapine (CLZ) [16]. We previously addressed this issue by showing that chemogenetic stimulation with a low dose of clozapine successfully induced neural responses with minimal off-target effects, thanks to the free passage of clozapine across the blood–brain barrier [17]. Further, low dose of CLZ can avoid fatal complications such as agranulocytosis and neutropenia [18].

In the current study, we tested the efficacy of clozapine-induced chemogenetic neuromodulation (CLZ-ChemoNM) in post-stroke recovery. We used the chronic capsular infarct model of stroke in rats, as described previously, and confirmed that motor deficits persisted for more than 2 weeks after the infarct [19, 20]. Our main goal was to evaluate the effect of excitatory CLZ-ChemoNM on recovery outcomes and to determine its translational feasibility. To this end, as well as behavior, we measured functional changes in the brain during chemogenetic neuromodulation using longitudinal 2-deoxy-2-[18F]-fluoro-D-glucose (FDG)-microPET imaging and identified neuroimaging biomarkers correlated with functional recovery. We also performed immunohistochemical studies to delineate the possible molecular mechanisms underlying post-stroke recovery. We propose that our combination of low-dose clozapine and hM3Dq-YFP DREADDs may be a good choice for translation of chemogenetic neuromodulation for recovery after stroke in humans.

## Materials and Methods

### Experimental Animals

Animal care and experimental procedures were approved by the Gwangju Institute of Science and Technology Animal Care and Use Committee. All experiments in the study were carried out in compliance with the ARRIVE guidelines. Experiments were performed on 36 male Sprague Dawley rats (9 weeks old, ~ 300 g). Rats were housed two per cage with ad libitum access to food and water. The animal care unit was maintained with constant temperature (21 °C ± 1 °C) and humidity (50%) and a 12-h light/dark cycle (07:00–19:00).

**Fig. 1:**
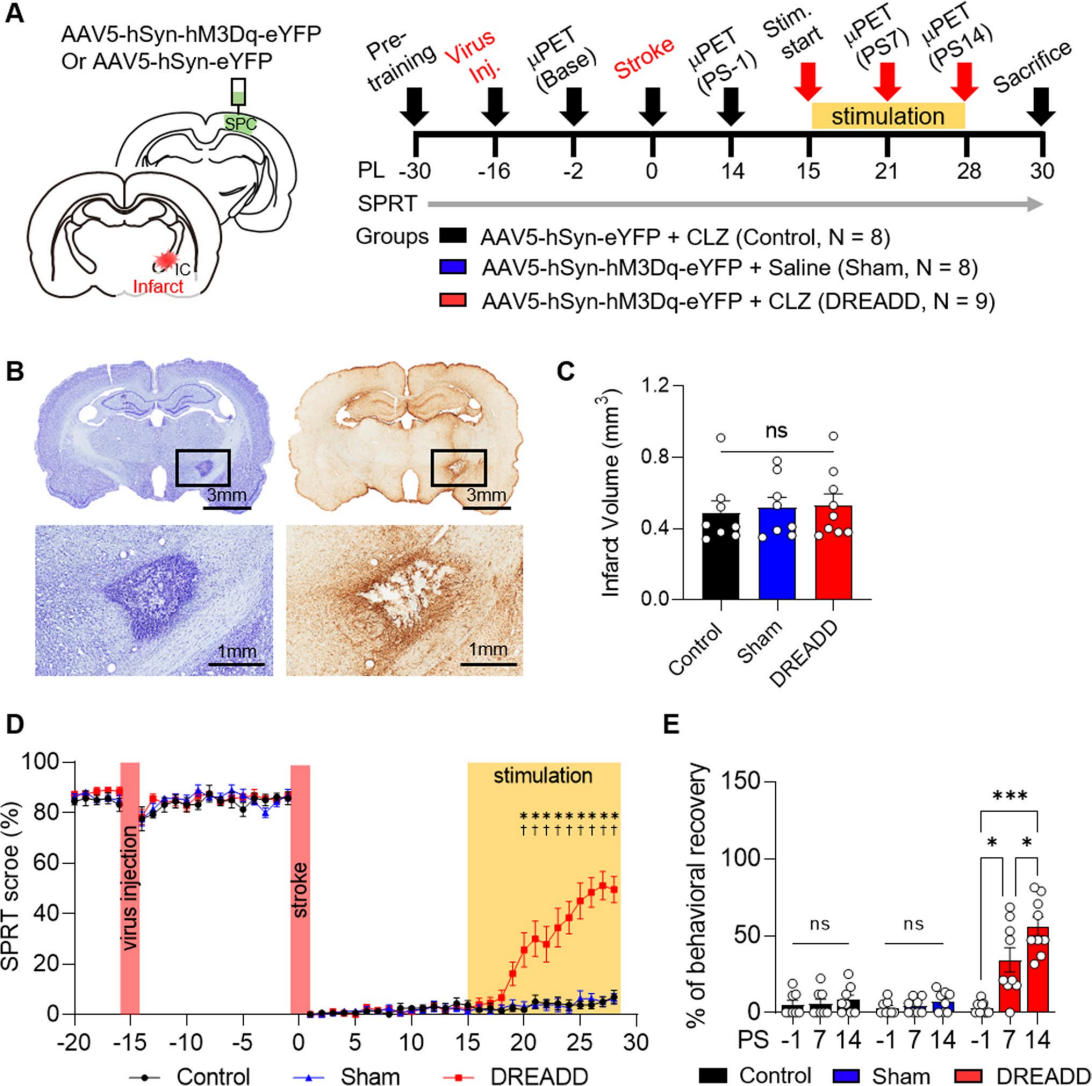
CLZ-ChemoNM of sensory-parietal cortex promotes functional recovery after chronic capsular infarct. **A** Animal groups and the experimental timeline. **B** Representative Nissl- (left) and GFAP- (right) stained sections of the internal capsule. **C** Volume of the infarct for the three different groups (*F*_(2,22)_ = 0.08747, *p* = 0.9166). **D** Daily performance in the single pellet reaching task (*F*_(92,1012)_ = 12.42, *p* < 0.0001). Yellow shading indicates the period of clozapine stimulation. **E** Behavioral recovery relative to pre-lesional SPRT performance (*F*_(4,44)_ = 20.11, *p* < 0.0001). Every point represents one animal. For all panels, data was represented as mean ± S.E.M., analyzed using one-way ANOVA with Tukey test (**C**) or RM two-way ANOVA with Bonferroni test (**D** and **E**) (*DREADD vs. control; ^†^DREADD vs. sham; *p < 0.05, ***p < 0.001; ^†^*p* < 0.05; ns, not significant)

For the electrophysiological verification of viral vectors, eleven rats underwent AAV5-hSyn-hM3Dq-eYFP (*N* = 5) or AAV5-hSyn-eYFP (*N* = 6) virus injection into the sensory-parietal cortex. For the investigation into the effect of DREADD-based chemogenetic neuromodulation on capsular stroke, twenty-five rats underwent AAV5-hSyn-hM3Dq-eYFP (DREADD group, *N* = 9; sham stimulation group, *N* = 8) or AAV5-hSyn-eYFP (control group, *N* = 8) virus injection into sensory-parietal cortex and photothrombotic capsular lesion of the posterior limb of the internal capsule (PLIC). Animals in the DREADD and control groups received clozapine (0.1 mg/kg, i.p.) during the stimulation period, whereas the sham stimulation group received saline (1 ml/kg, i.p.) (Fig. 1A).

### Viral Vector Injection

All animals were anaesthetized with a mixture of ketamine/xylazine and fixed in a stereotaxic apparatus. We used excitatory hM3Dq DREADDs (AAV5-hSyn-hM3Dq-eYFP) for chemogenetic neuromodulation, which is known to be expressed mainly at the plasma membrane and control virus (hSyn-hM3Dq-eYFP). A small craniotomy was made, and 1 µl of AAV5-hSyn-hM3Dq-eYFP (KIST virus facility, Seoul, Korea) or AAV5-hSyn-eYFP (KIST virus facility, Seoul, Korea) was injected into the sensory-parietal cortex (coordinates from bregma: AP =  − 4.0 mm, ML =  ± 3 mm, DV =  − 1 mm) at a rate of 0.1 ml/min using a 33G NanoFil syringe connected to an UltraMicroPump (WPI, FL, USA). After injection, the needle was left in place for an additional 10 min before being slowly retracted. After the scalp wound was sutured, postoperative pain was controlled with ketoprofen (2 mg/kg, i.m.).

### Electrophysiological Verification of Viral Vectors

We used electrophysiology to verify changes in spike firing in neurons where AAV5-hSyn-hM3Dq-eYFP and hSyn-hM3Dq-eYFP were injected. Animals were fixed in a stereotactic frame under urethane anesthesia (1.5 g/kg, i.p.) A craniotomy was made over the region of viral expression (coordinates from bregma: AP =  − 4.0 mm; ML =  ± 3 mm). Then, the exposed area was covered with mineral oil to prevent drying. A 16-channel microelectrode array (NeuroNexus, MI, USA; A1 × 16 100 µm site spacing) was slowly inserted at the target site. Raw signals were band-pass filtered (250 to 6000 Hz), amplified (20 × 1000), and digitized at 40 kHz by an OmniPlex system with PlexControl software (Plexon, Dallas, TX). After 30 min of basal recording, each rat received a single dose of clozapine (0.1 mg/kg, i.p.). Spike detection and sorting were performed in Offline Sorter (Plexon, Dallas, TX). The spike threshold level was set to 5.5 × the SD (standard deviation) of each signal. The average firing rates were calculated as the average frequency of spike firing during the 30-min periods before and after clozapine injection. After recordings were completed, the rats were perfused transcardially with 0.9% saline solution followed by 4% paraformaldehyde (PFA) in 0.1 M phosphate-buffered saline (PBS). Then, the brains were sectioned coronally at 40-µm thickness to confirm viral expression and the location of the electrode tract.

### Photothrombotic Capsular Infarction

Two weeks after the viral injection, animals underwent photothrombotic infarct lesioning in the PLIC as described in previous studies [19, 20]. Briefly, rats were anesthetized with a mixture of ketamine/xylazine and mounted on a stereotaxic apparatus. After a scalp incision along the midline, a small craniotomy was made and an optical fiber (62.5-μm core diameter and 125-μm cladding diameter) was stereotaxically inserted into the PLIC (coordinates from bregma: AP = 2.0 mm, ML =  ± 3.1 mm, DV = 7.8 mm). Rose Bengal dye (20 mg/kg) was injected through the tail vein. Then, the target was irradiated with a green laser (3.7 mW) for 1.5 min. After the optical fiber was removed, the scalp wound was secured and treated with ketoprofen (2 mg/kg, i.m.) for postoperative pain control.

### Behavioral Testing

The single-pellet reaching task (SPRT) was daily performed to evaluate skilled motor behavior throughout the experimental period [21, 22]. Rats were food-restricted to 90% of their initial body weight to motivate food retrieval. Rats were placed inside a clear Plexiglas (45 cm × 40 cm × 13 cm) box with a 1-cm wide slit and food shelf in the midline of the front wall. During the pre-training period, the preferred handedness of each rat was determined by evaluating how successful the preferred paw was in retrieving sucrose pellets (Bio-Serve, Frenchtown, NJ) that had been placed obliquely on the shelf. A reach was considered successful if the rat extended its preferred forelimb to grasp the pellet on the shelf and brought the pellet into its mouth without dropping it. Rats received 20 pellets per session, with each session lasting for 20 min. The reaching score was calculated as follows:

$$\frac{\mathrm{Number}\;\mathrm{of}\;\mathrm{successful}\;\mathrm{reaches}\;\times\;100}{20}$$

### Ligand Administration for Chemogenetic Stimulation

Clozapine (Tocris Bioscience, Bristol, UK) was initially dissolved in DMSO and then diluted to a final concentration of 0.1 mg/ml clozapine in 3% DMSO solution in saline solution. For chemogenetic neuromodulation of the sensory cortex, rats were injected (i.p.) with 0.1 mg/kg clozapine (DREADD group and control group) or 1 ml/kg saline (sham stimulation group) once daily from PL 15 to PL 28 after capsular infarct surgery.

### MicroPET Image Acquisition and Processing

Longitudinal microPET scans were performed to investigate changes in regional glucose metabolism and cortical diaschisis before and after chemogenetic stimulation. Rats underwent a total of four scanning sessions: the first scan was performed prior to the infarct lesioning (baseline scan), the second scan was performed 2 weeks after the infarct lesioning (PS-1, i.e., just before the start of chemogenetic stimulation), and the third and fourth scans were performed 7 and 14 days after chemogenetic stimulation (PS7 and PS14).

Animals were fasted for 12 h to attain consistency in blood glucose levels prior to scanning. The rats were injected with clozapine (0.1 mg/kg) or saline (1 ml/kg) intravenously under brief isoflurane anesthesia (1.5%). Thirty minutes after drug administration, the rats were injected intravenously with 18F-FDG (0.1 mCi/100 g). After a thirty-minute uptake period, rats were anesthetized with 2% isoflurane and placed in a prone position on the microPET scanner (Siemens Medical Solutions, TN, USA). A 25-min static PET acquisition and 5-min attenuation-correction CT scan were performed. During the scanning, vital signs were monitored (BioVet; m2m Imaging Corp, Newark, NJ, USA), including respiration (50 ± 5 respirations/min), heart rate (280 ± 20 beats/min), and body temperature (37.0 ± 1 °C). The acquired images were corrected for attenuation and reconstructed with the 3-dimensional ordered-subsets expectation maximum (3D-OSEM) algorithm with scatter correction and random correction.

Image analysis was performed with the Analysis of Functional NeuroImages (AFNI) package [23]. All acquired PET images were co-registered and spatially normalized to the MRI template [24]. Then, each image was normalized to the mean value of the whole brain and spatially smoothed with an isotropic Gaussian kernel with 1.2 mm full width at half maximum. The MRIcroGL program was used to create the 3-D rendered images (https://www.nitrc.org/projects/mricrogl/).

### Histological Examination

After the last microPET/CT scan was complete, animals received clozapine (0.1 mg/kg) or saline (1 ml/kg). Ninety minutes later, animals were perfused with 0.9% saline followed by 4% paraformaldehyde (PFA) under ketamine anesthesia (100 mg/kg body weight). Brains were post-fixed overnight in 4% PFA and cryoprotected in 30% sucrose in phosphate buffered saline. Then the brains were serially sectioned into 40-µm sections at 200-µm intervals. Nissl and anti-GFAP staining (1:300, Millipore, AB5541) was used to confirm the circumscribed capsular infarct and to measure infarct volume. c-Fos staining (1:1000, Cell Signaling, 2250S) was used as a marker of neuronal activity. Images were acquired on an Olympus VS200 slide scanner (SLIDEVIEW VS200, Olympus, Tokyo, Japan) with 20 × (UPLXAPO20X, NA = 0.8) air objective lens.

Fluorescent immunohistochemistry was performed on the brain sections. Anti-GFP (1:1000, Abcam, ab1218) and anti-NeuN (1:1000, Millipore, ABN90P) staining was performed to confirm viral expression. Anti-BDNF (1:500, Alomone Labs, ANT-010) was used to detect this activity-dependent neurotrophic factor. Fluorescent secondary antibodies were purchased from Invitrogen and used in 1:200 dilutions. Fluorescence images (1024 × 1024) were acquired on an Olympus FV3000 confocal laser scanning microscope (FLUOVIEW FV3000, Olympus, Tokyo, Japan) with 20 × (UPLXAPO20X, NA = 0.8) and 40 × (UPLXAPO40X, NA = 0.95) air objectives equipped with solid state lasers (488, 555, and 647 nm) at an exposure of 8 µs/pixel and a numerical zoom of 1 × .

For c-Fos quantification, each image was converted to an 8-bit image, and the despeckle function was applied to reduce noise. Next, images were thresholded with ImageJ (https://imagej.nih.gov/ij/) so that only c-Fos-positive cells remained. A custom MATLAB-based program (MathWorks, Natick, MA, USA) was used to create c-Fos density maps from the thresholded images. The number of c-Fos-positive cells was counted in eight ROIs (1 mm × 1 mm), including bilateral motor cortex, cingulate gyrus, sensory cortex, and striatum, that showed marked changes in c-Fos expression after chemogenetic stimulation.

To analyze BDNF intensity in NeuN-positive cells, each image was first converted to an 8-bit image, and NeuN-positive images were converted into binary images. The binary NeuN-positive images and BDNF-positive image were multiplied so that the BDNF signal remained only in pixels that were also positive for NeuN. In the multiplied images, the mean intensity value of BDNF was measured in each ROI.

### Statistical Analysis

For the FDG-microPET statistical mapping, a group-level linear mixed-effect model was performed with the 3dLME program in AFNI. The image analysis compared pre-lesional (base) and post-lesional images (PS-1, PS7 and PS14) to assess time-dependent changes in cortical diaschisis and regional glucose metabolism. Statistical maps were thresholded at the significance level (*p* < 0.001, false discovery rate *q* < 0.05). To measure the longitudinal changes in normalized mean activity (NMA) of cortical diaschisis area, ROI mask was created based on statistically thresholded maps of PS-1 (*p* < 0.001) in each group. Additional image analysis compared the pre-stimulation (PS-1) and post-stimulation (PS7 and PS14) images to assess the effects of clozapine-induced chemogenetic stimulation. Statistical maps were thresholded at the significance level (*p* < 0.01), and the results were corrected for multiple comparisons with the 3dClustSim program in AFNI (*α* = 0.05, *p* < 0.01, *k* < 39). Six regions of interest were also defined manually in bilateral motor cortices, sensory cortices, and the striatum. All statistical maps were overlaid on the MRI template to show regions of significant metabolic change.

Data were analyzed in Prism 7 (GraphPad). Electrophysiology, time-dependent changes in cortical diaschisis volume, NMA, and SPRT performance data were analyzed with a repeated-measures two-way ANOVA with Bonferroni post hoc test. In addition, c-Fos data was analyzed with two-way ANOVA with Bonferroni post hoc test. Infarct volume and BDNF data were analyzed with a one-way ANOVA with Tukey test. Linear regression (*p* < 0.05) was used to measure the correlations between NMA and SPRT scores and between the changes in NMA or diaschisis volume and SPRT scores. All data are represented as the mean ± standard error of the mean (S.E.M.). The significance level is represented by asterisks (*p < 0.05, **p < 0.01, ***p < 0.001; ns, not significant).

## Results

### CLZ-ChemoNM Induces a Neural Response

**Fig. 2:**
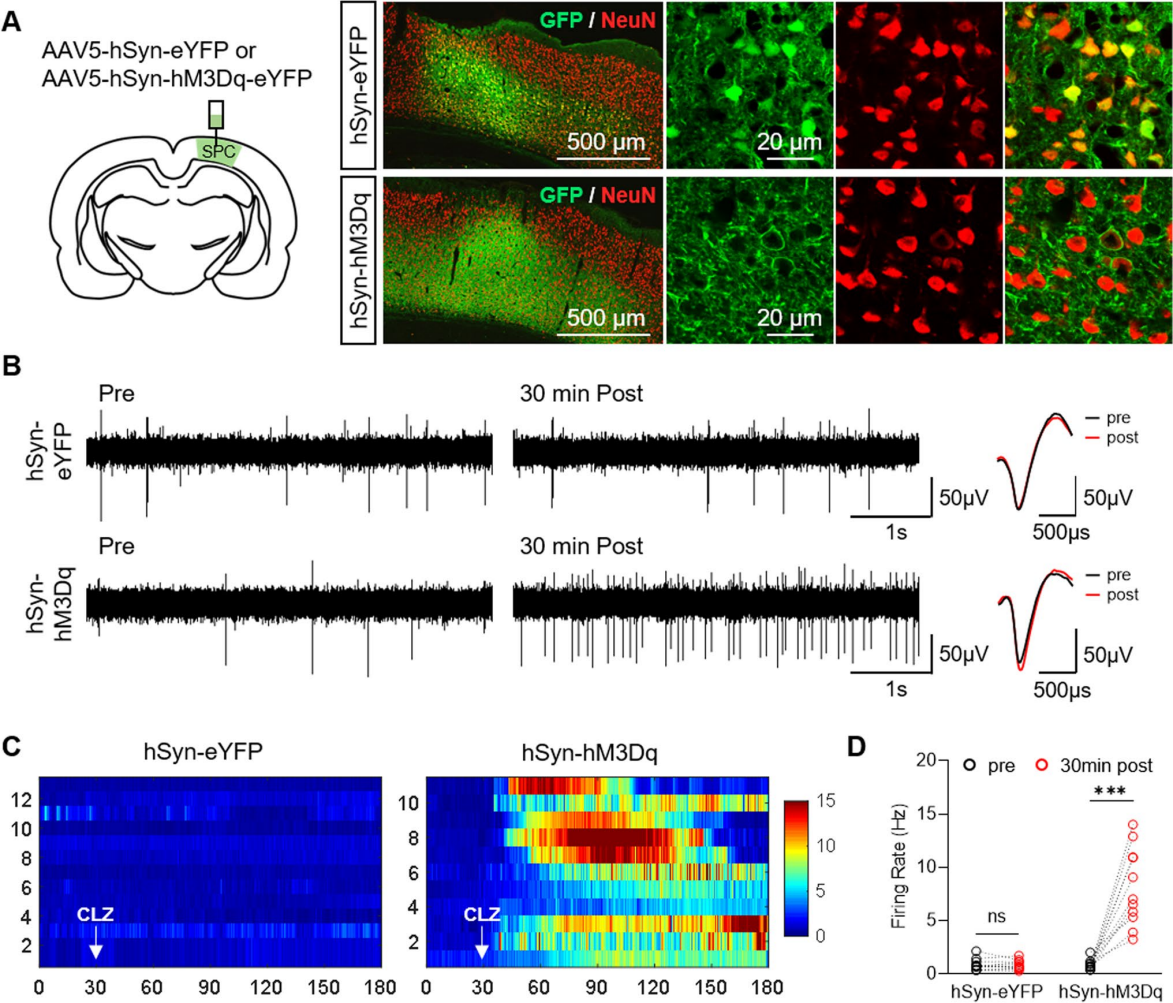
CLZ-ChemoNM induces a prolonged neural response. **A** Schematic diagram of the viral injection and representative images of viral expression in the sensory-parietal cortex (SPC). Brain sections were co-stained for GFP and NeuN. **B** Representative extracellular trace and spike waveform before (pre) and 30 min after (post) clozapine administration (0.1 mg/kg, i.p.). **C** Spike raster plots of neuronal responses from the virus-injected region after clozapine administration. Numbers on the *Y*-axis indicate individual recorded units (*N* = 12 and 11 for hSyn-eYFP and hSyn-hM3Dq, respectively). The scale bar indicates the frequency of neuronal firing (Hz). **D** Comparison of firing rates before and 30 min after clozapine administration (*F*_(1,21)_ = 42.49, *p* < 0.0001). Individual points in **D** represent each recorded unit. Data was analyzed using RM two-way ANOVA with Bonferroni test (d) (***p < 0.001; ns, not significant)

First, we used electrophysiology to test whether CLZ-ChemoNM could provoke a neuronal response sufficient to influence the relevant neural circuits. We transfected AAV5-hSyn-eYFP (control) or the Gq-coupled excitatory DREADD virus, AAV5-hSyn-hM3D(Gq)-eYFP in rat somatosensory cortex (Fig. 2A). We performed extracellular recordings using a 16-channel electrode introduced into the virus-expressing area under urethane anesthesia. CLZ (0.1 mg/kg, i.p.) injected had no detectable effect on firing in the control group; however, it elicited a marked increase in firing rate in the DREADD group (Fig. 2B). In DREADD-expressing animals, the spike firing rate in individual cells doubled between 6 and 20 min after CLZ injection, and the increase persisted for at least 89 min (Fig. 2C). When firing rates were compared before (pre) and 30 min after CLZ injection, only neurons recorded from DREADD-expressing animals showed a significant increase (*p* < 0.001) in firing rate (Fig. 2D). These results demonstrate that CLZ-ChemoNM elicited a prolonged neural response.

### CLZ-ChemoNM Enhances Post-stroke Recovery in a Chronic Capsular Infarct Model

We asked whether CLZ-ChemoNM could promote behavioral recovery following capsular infarct. All animals underwent stereotactic photothrombotic lesioning to model capsular infarct, as described in our previously established protocol [19, 20]. This technique generates a circumscribed infarct lesion in the internal capsule, leading to persistent motor impairment of the forelimb. Animals were divided into three groups: (1) the experimental DREADD group, which received injection of the Gq-coupled excitatory DREADD virus AAV5-hSyn-hM3D(Gq)-eYFP in the sensory-parietal area and subsequent CLZ injection (0.1 mg/kg) to activate the transfected neurons; (2) a control group, which received injection of a control virus (AAV5-hSyn-eYFP) and CLZ injection (0.1 mg/kg, i.p.); and (3) a sham-operated group, which received injection of the DREADD virus AAV5-hSyn-hM3D(Gq)-eYFP but saline injection instead of CLZ. For all animals, the virus injection was performed 2 weeks before infarct lesioning, to guarantee sufficient time for viral expression (see Fig. 1A for the full experimental time line). Animals in all of the experimental groups had lesions with similar infarct volumes (Fig. 1B and C). Rats performed the single-pellet reaching task (SPRT) for 20 min daily [22]. All groups showed an immediate, persistent decline in reaching performance following infarct lesioning. After confirming that the motor impairment persisted for at least for 2 weeks, we administered CLZ-ChemoNM and found that the SPRT scores improved significantly in the experimental DREADD group after 4 days of neuromodulation, eventually reaching 56% of the pre-infarct score (Fig. 1D and E). By contrast, there was no improvement in either the control or sham-operated groups, despite all groups receiving the same amount of daily SPRT training. These results suggest a causal relationship between CLZ-ChemoNM and post-infarct behavioral recovery.

### CLZ-ChemoNM Induces Functional Changes in Regional Glucose Metabolism After Chronic Capsular Infarct

Though CLZ-ChemoNM provides cell-specific stimulation, it is unclear how specific brain circuits or areas are recruited to promote behavioral recovery. After a stroke, functional changes occur in brain regions remote from the focal lesion. These changes are known as diaschisis and can be used as a marker of post-stroke recovery after capsular infarct [25, 26]. Previously, we reported that electrical stimulation and intensive rehabilitative training reduced the volume of diaschisis and were significantly correlated with improvements in post-stroke motor impairments [26, 27]. We also showed that diaschisis after capsular infarct is caused by GABA-synthesizing reactive astrocytes in cortical areas distant to the lesion, leading to tonic inhibition of the neighboring neurons [25].

**Fig. 3:**
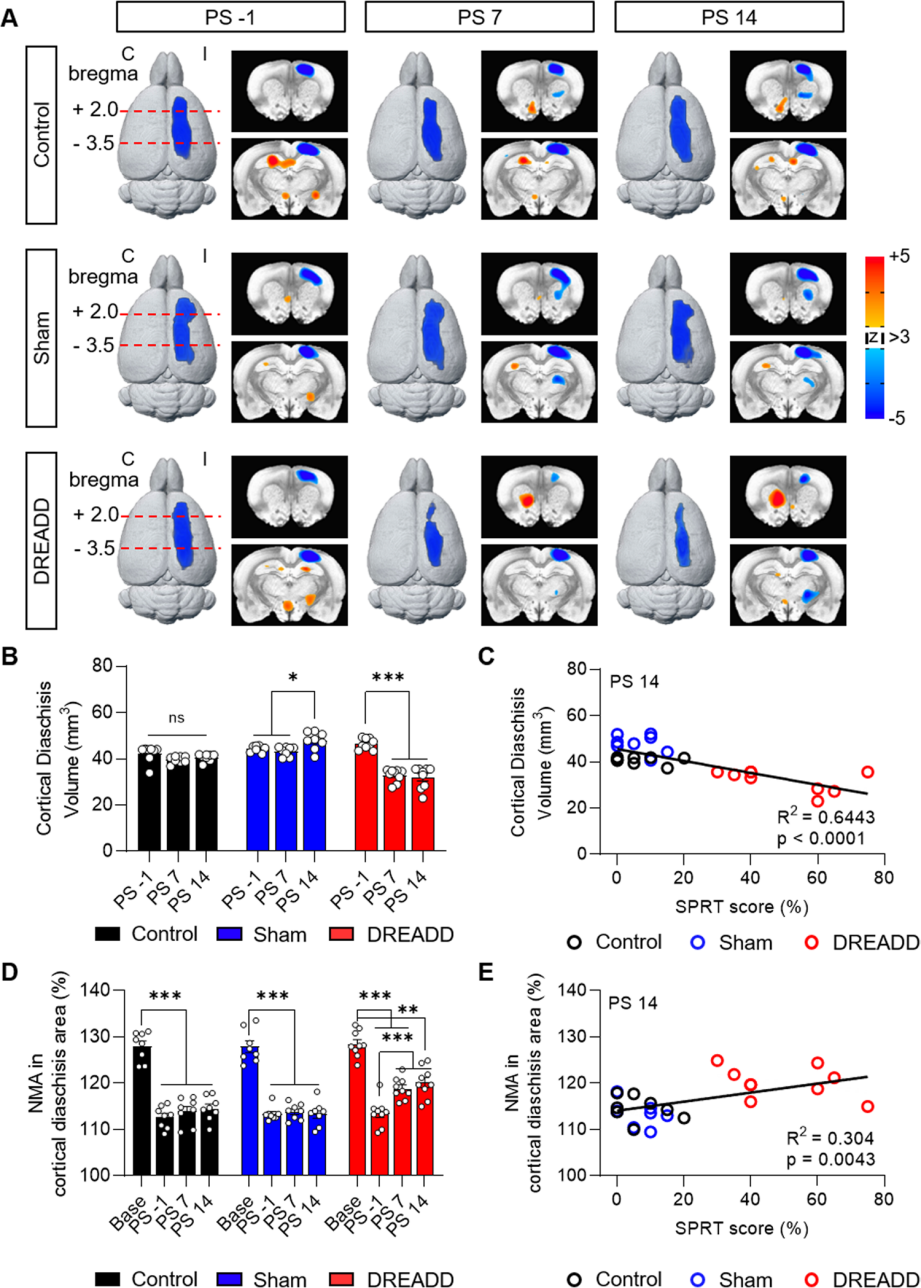
CLZ-ChemoNM reverses cortical diaschisis in a chronic stroke model. **A** 3-D rendered images of FDG-microPET scans showing longitudinal changes in cortical diaschisis in each group (*N* = 8, 8, and 9 for control, sham, and DREADD groups, respectively). Blue regions indicate a significant reduction in glucose metabolism. Image analysis comparing the pre-lesional (base) and post-lesional (PS-1, PS7 and PS14) images was performed to assess time-dependent changes in cortical diaschisis (3dLME in AFNI, *p* = 0.001, false discovery rate *q* < 0.05). C, contralesional; I, ipsilesional. **B** Volume of cortical diaschisis (*F*_(4,44)_ = 60.04, *p* < 0.0001). **C** At PS14, cortical diaschisis volume was negatively correlated with SPRT performance (*F*_(1,23)_ = 41.66). **D** Normalized mean activity (NMA) in the cortical diaschisis area. The DREADD group showed a significant increase in NMA after CLZ-ChemoNM (*F*_(6,66)_ = 6.877, *p* < 0.0001). **E** At PS14, the NMA in the cortical diaschisis region was positively correlated with SPRT performance (*F*_(1,23)_ = 10.05). Every point represents one animal. For all panels, data was represented as mean ± S.E.M., analyzed using RM two-way ANOVA with Bonferroni test (**B** and **D**) or linear regression (**C** and **E**) (*p < 0.05, **p < 0.01, ***p < 0.001; ns, not significant)

To identify the circuits and regions responsible for neuromodulation-induced behavioral recovery, we measured the effects of CLZ-ChemoNM on diaschisis using longitudinal FDG-microPET scans. All animals were scanned a total of four times: a baseline scan after preoperative training and viral injection but before infarct lesioning (base), a scan 14 days post-lesion, just prior to the start of CLZ-ChemoNM (PS-1), and two scans 7 and 14 days after the start of CLZ-ChemoNM (PS7 and PS14, respectively). To delineate the diaschisis clearly, we performed an image analysis, comparing the pre-lesional and post-lesional longitudinal images. The volume of cortical diaschisis was significantly reduced in the DREADD group at PS7 and PS14, and this reduction was significantly correlated with the improved SPRT scores, in agreement with previous findings (Fig. 3A-C). The control group did not show a significant reduction in the volume of cortical diaschisis, and the sham-operated group showed an increase in the volume of diaschisis between PS7 and PS14 (Fig. 3A–C and Fig. S1). The normalized mean activity (NMA) decreased significantly relative to baseline in both the control and sham-operated groups, indicating the strength of the diaschisis, but the NMA increased progressively and significantly in the experimental DREADD group over the stimulation period (Fig. 3D). The increased NMA was positively correlated with improved SPRT scores (Fig. 3E). These data suggest that CLZ-ChemoNM played a causal role in reducing the volume of diaschisis and increasing the NMA and that this may have contributed to the reversal of the post-stroke motor deficit.

**Fig. 4:**
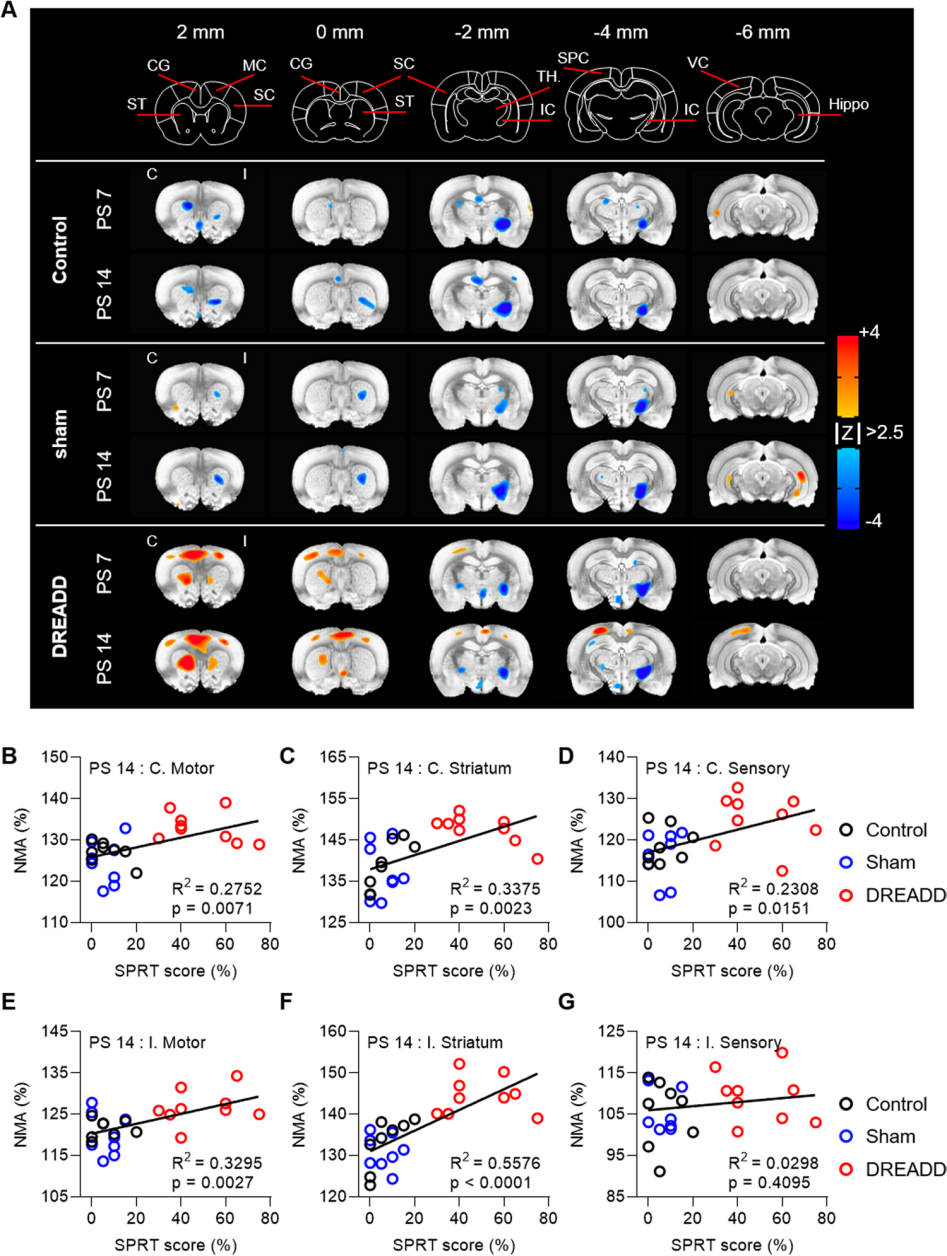
Distinct involvement of corticostriatal circuits in post-stroke recovery following chemogenetic stimulation. **A** Color-coded maps show the activated and deactivated regions in each group (*N* = 8, 8, and 9 for control, sham, and DREADD groups, respectively). Image analysis comparing the pre-stimulation (PS-1) and post-stimulation (PS 7 or PS 14) images was performed to assess the effect of CLZ-ChemoNM (3dClustSim in AFNI *p* < 0.01, *α* = 0.05, *k* < 39). CG, cingulate gyrus; ST, striatum; MC, motor cortex; SC, sensory cortex; IC, internal capsule; TH, thalamus; SPC, sensory-parietal cortex; Hippo, hippocampus; C, contralesional; I, ipsilesional. **B–G** Linear regression between SPRT score and normalized mean activity (NMA) in each ROI. At PS14, NMA in bilateral motor cortex and striatum and contralateral sensory cortex, but not ipsilateral sensory cortex, was positively correlated with SPRT performance (**B**, *F*_(1,23)_ = 8.732; **C**, *F*_(1,23)_ = 11.72;. **D**, *F*_(1,23)_ = 6.889; **E**, *F*_(1,23)_ = 11.30; **F**, *F*_(1,23)_ = 28.99; **G**, *F*_(1,23)_ = 0.7059). Every point represents one animal. Data was analyzed using linear regression (**B–G**)

To further probe the effect of chemogenetic stimulation, we performed image analysis of the FDG-microPET scans taken pre- and post-stimulation. When comparing the experimental group with the control and sham-operated groups, increased NMA was significantly prominent in bilateral cortices, including motor, cingulate, and contralesional sensory cortex, and in the striatum (Fig. 4A). Interestingly, activation was more prominent on the contralesional side than on the ipsilesional side, where stimulation was applied. The increased NMA was positively correlated with SPRT scores (Fig. 4B–F). In the ipsilesional sensory cortex, however, we could not observe neither alteration of NMA nor the correlation between NMA and SPRT scores (Fig. 4G). As shown in our previous work, activation of DREADDs by clozapine did not produce an increase in regional glucose metabolism in the ipsilesional sensory cortex [17]. These data indicate that CLZ-ChemoNM in the sensory-parietal cortex mainly activates bilateral areas in the corticostriatal circuit, contributing to an increase in reaching performance in the DREADD group compared with the control and sham-operated groups.

### CLZ-ChemoNM Induces Widespread c-Fos Expression

**Fig. 5:**
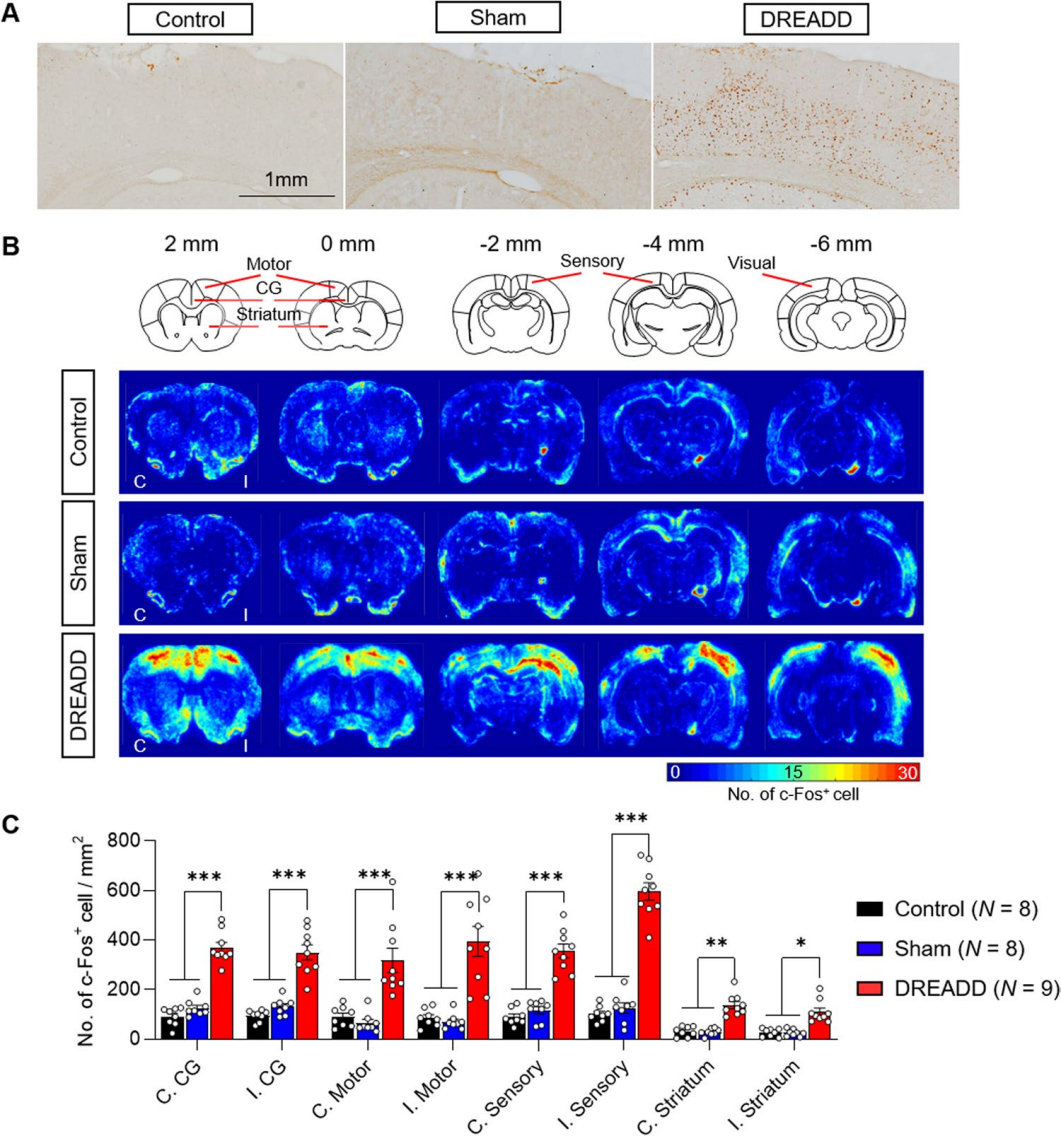
CLZ-ChemoNM induces widespread c-Fos expression. **A** Representative c-Fos expression in ipsilesional sensory cortex. **B** Density map of c-Fos expression for each group. Note that only the DREADD group shows widespread expression of c-Fos following clozapine stimulation. CG, cingulate gyrus; C, contralesional; I, ipsilesional. **C** Quantification of c-Fos-positive cells in bilateral cingulate gyrus, motor cortex, sensory cortex, and striatum for each group (*F*_(14,176)_ = 10.03, *p* < 0.0001). Every point represents one animal. Data was represented as mean ± S.E.M. and analyzed using two-way ANOVA with Bonferroni test (**C**) (*p < 0.05, **p < 0.01, ***p < 0.001)

Because long-term brain stimulation can influence distributed brain areas [28], we mapped the expression of c-Fos protein, a marker of neuronal depolarization, to determine the extent of brain activation at the cellular level following long-term CLZ-ChemoNM. Although CLZ-ChemoNM was performed in unilateral sensory-parietal cortex, c-Fos expression extended bilaterally, including motor, sensory, and cingulate cortices and the striatum (Fig. 5). This pattern is similar to that seen after chronic unilateral electrical stimulation of the cortex, which increases c-Fos expression contralaterally as well as ipsilaterally [28]. Given that increased spontaneous firing in somatosensory cortex leads to the expression of immediate early genes (e.g., c-fos) and is a prime candidate mechanism for modulating and maintaining neural connectivity [29–31], our results suggest that unilateral cortical stimulation can enforce stimulation-dependent neural connectivity in both hemispheres, ultimately contributing to the enhancement of post-stroke recovery.

### ChemoNM Enhances Neural Plasticity After Chronic Capsular Infarct

**Fig. 6:**
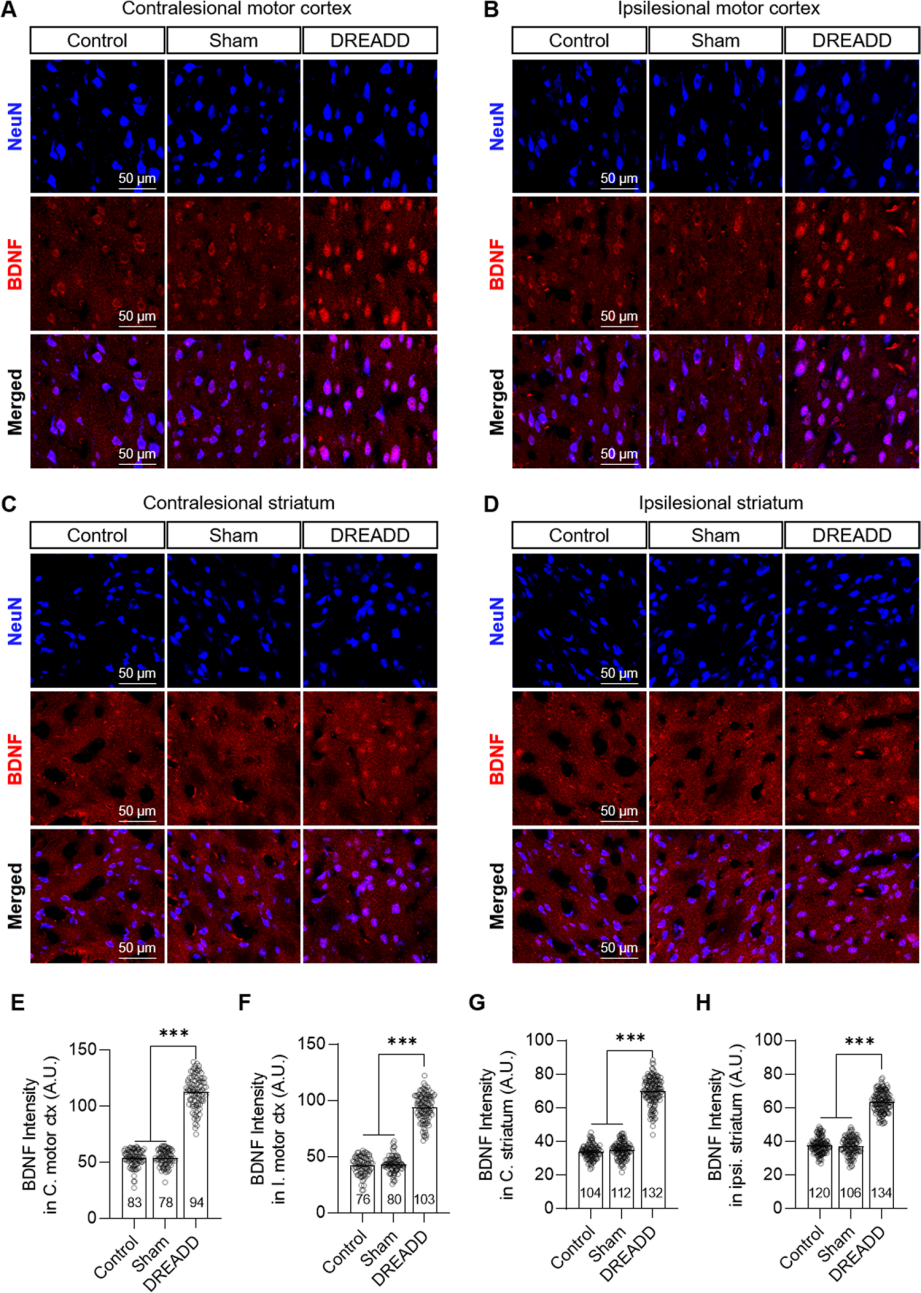
CLZ-ChemoNM increases BDNF expression. **A–D** Representative confocal images of NeuN and BDNF staining in bilateral motor cortex and striatum. **E–H** Quantification of BDNF intensity for each ROI (one image from one animal, *N* = 4, 4, and 5 for control, sham, and DREADD groups, respectively). Animals in the DREADD group exhibit significantly higher BDNF expression in bilateral motor cortex and striatum (**E**, *F*_(2,252)_ = 24.78, *p* < 0.0001; **F**, *F*_(2,256)_ = 14.89, *p* < 0.0001; **G**, *F*_(2,345)_ = 24.23, *p* < 0.0001; **E**, *F*_(2,357)_ = 5.162, *p* = 0.0062). The number on each bar refers to the number of cells analyzed. Data was represented as mean ± S.E.M., analyzed using one-way ANOVA with Tukey test (**E**–**H**) (***p < 0.001)

Next, we sought to determine whether CLZ-ChemoNM enhances expression of brain-derived neurotrophic factor (BDNF), which plays a role in activity-dependent neural plasticity, including upregulation of synaptic proteins and alteration of synaptic properties [31–33]. We measured BDNF expression in bilateral motor cortex and the striatum, the regions that showed a prominent increase in regional glucose metabolism in the FDG-microPET experiment. The DREADD group, but not the control or sham-operated groups, showed a significant increase in BDNF expression intensity (Fig. 6). In addition, Increased BDNF expression in the DREADD-expressing neurons was observed after CLZ-ChemoNM (Fig. S2). These data suggest that BDNF-mediated neural plasticity is a major driver of the neuronal plasticity that leads to post-stroke recovery with CLZ-ChemoNM.

## Discussion

Our results demonstrate that a low dose of clozapine successfully activates DREADD receptors, leading to prolonged stimulation of the target brain area with minimal off-target effects [17]. Importantly, CLZ-ChemoNM enabled recovery from a chronic motor deficit (i.e., a deficit that persisted for more than 14 days after the infarct); such deficits tend not to respond easily to other neuromodulatory methods [7]. The effect of CLZ-ChemoNM was consistent with the effects of other neuromodulatory methods used in our previous studies to restore post-stroke function [25, 27]. Furthermore, CLZ-ChemoNM produced functional changes in microPET images of the brain, in agreement with our previous findings that behavioral recovery is correlated with a reduction in diaschisis volume and activation of a corticostriatal recovery circuit [25, 26]. We thus suggest that CLZ-ChemoNM has the potential to replace conventional electrical stimulation and other neuromodulatory methods to reverse post-stroke deficits [12].

Despite the promising potential of chemogenetic neuromodulation, there are two critical factors that need to be considered before the technique can be translated to humans: the safety and transduction efficiency of the virus and the pharmacodynamics of the DREADD agonist. First, although the use of rAAV gene therapy is increasing, there are limitations of this gene delivery platform including rAAV manufacturing and immunological barriers [34]. Secondly, it is ideal to make the DREADDs that are expressed in the plasma membrane of the targeted cells, where G-protein-coupled receptors are functionally linked to their intracellular signaling pathways and can respond to exogenous ligands [14, 15]. A recent ultrastructural study revealed structural differences between the inhibitory hM4Di and excitatory hM3Dq DREADDs, leading to discrepant patterns of receptor localization in the plasma membrane versus the intracellular compartment: hM4Di is poorly transported to plasma membrane in monkeys despite being abundantly expressed in the membrane in mice, whereas hM3Dq is expressed mainly at the plasma membrane in both monkeys and mice [35]. In this regard, we used hM3Dq-YFP for neuromodulation because it is expressed in the plasma membrane, leading to greater potential to facilitate a robust and consistent effect.

Regarding agonist pharmacodynamics, we chose to use a low dose of clozapine to evoke chemogenetic activation over a prolonged duration. In our previous study, we confirmed that clozapine elicits a neural response within 30 min of administration in DREADD-expressing rats. We also demonstrated that a low dose of clozapine (0.1 mg/kg) is sufficient to elicit a neural response and reduces off-target effects [17]. If a more prolonged period of excitability is needed, the frequency of administration of CLZ can be increased. Clozapine is also an FDA-approved drug and is currently prescribed to treat drug-resistant schizophrenia [36]. Therefore, despite recent reports of chemogenetic perturbation of inhibitory or glutamatergic neurons with CNO in rodents [37, 38], our results provide a road-map for overcoming the major hurdles in translating chemogenetic neuromodulation to human patients.

When a new treatment strategy is applied to manage stroke disorders, it is critical to have a sensitive and specific biomarker to predict the outcome and progress of that treatment. Our results demonstrate that functional brain imaging, such as microPET, can help elucidate neuromodulation-induced functional changes in brain regions and circuits and link these changes to the final outcome. Previous reports have consistently demonstrated a time-dependent increase in diaschisis in the cortical areas remote to the site of injury, linked to ultrastructural dendritic changes [39]. In addition, we reported that GABA-synthesizing reactive astrocytes in distant cortical areas cause glucose hypometabolism via tonic inhibition of neighboring neurons, leading to a diaschisis [25]. MAO-B inhibition causes a significant reduction of diaschisis and an enhanced post-stroke recovery when combined with a rehabilitation regimen. In line with such findings, we found that CLZ-ChemoNM reduced the volume of diaschisis and activated a corticostriatal circuit. We also investigated the effect of CLZ-ChemoNM on plasticity by examining the expression of the activity-dependent neurotrophin, BDNF. Animals in DREADD group exhibit significantly higher BDNF expression than control and sham group, suggesting brain plasticity is contributing to post-stroke recovery [31–33]. Our results suggest that these changes may serve as a useful imaging biomarker to predict stroke recovery outcomes after rehabilitation, electrical cortical stimulation, and/or pharmacological treatments [25–27]. Notably, the neuromodulation-induced effects were more prominent in the hemisphere contralateral to DREADD expression than in the ipsilateral hemisphere. Clozapine is known to influence metabolic activity in multiple areas of the brain, including the motor and sensory cortices [40, 41]. Although we used a low dose of clozapine, we cannot rule out the possibility that clozapine depressed cortical metabolism directly, which could lead to a decreased reduction in diaschisis and reduced activation of the corticostriatal circuit. Future studies are needed to further elucidate the role of clozapine in CLZ-ChemoNM.

There is concern that the window of opportunity for rehabilitation may not be substantially longer in humans than in smaller species [42], and rehabilitation beyond this standard therapeutic time window remains a major challenge in post-stroke survivors [3, 43]. Most stroke research has focused on therapeutic improvements in acute models of stroke. However, in this study, we used our chronic capsular infarct model, which produces motor deficits that persist for more than 3 months, and performed chemogenetic neuromodulation at least 2 weeks after infarct lesioning. Our results demonstrate that CLZ-ChemoNM successfully induced a variety of stroke recovery signals during the chronic stage of stroke. Nonetheless, the time window of therapeutic opportunity in animal models is limited and not consistent between species [44, 45]; thus, the mechanism by which CLZ-ChemoNM operates beyond this time window needs to be further elucidated.

The plethora of new treatment methods for stroke disorders that have failed during clinical trials has raised concerns about how best to perform preclinical studies to assess the translational feasibility of such interventions. Stroke research in small animals may not translate well to humans because of the barriers imposed by anatomical and physiological differences between the species [46]. Non-human primates have similar gyrencephalic structures as humans and can help bridge this gap [47]. Although we have demonstrated the efficacy of CLZ-ChemoNM for rescuing post-stroke deficits in rats, our results should be replicated in non-human primates before translation to humans.

## Supplementary Information

Below is the link to the electronic supplementary material.

Supplementary file1 (DOCX 1316 KB)

## Data Availability

All relevant data are available from the authors upon reasonable request.

## References

[1] Collaborators GBDN (2019). Global, regional, and national burden of neurological disorders, 1990–2016: a systematic analysis for the Global Burden of Disease Study 2016. Lancet Neurol.

[2] Salim S. Virani AA, Hugo J. Aparicio, Emelia J. Benjamin, Marcio S. Bittencourt, Clifton W. Callaway, April P. Carson,. Heart disease and stroke statistics-2021 update: a report from the American Heart Association. Circulation. 2021;143:e254-e743. 10.1161/CIR.0000000000000950 PMC13036842 33501848

[3] Avan A, Digaleh H, Di Napoli M, Stranges S, Behrouz R, Shojaeianbabaei G (2019). Socioeconomic status and stroke incidence, prevalence, mortality, and worldwide burden: an ecological analysis from the Global Burden of Disease Study 2017. BMC Med.

[4] Dromerick AW, Geed S, Barth J, Brady K, Giannetti ML, Mitchell A, et al. Critical Period After Stroke Study (CPASS): a phase II clinical trial testing an optimal time for motor recovery after stroke in humans. Proc Natl Acad Sci USA. 2021;118. 10.1073/pnas.2026676118 PMC8488696 34544853

[5] Dawson J, Liu CY, Francisco GE, Cramer SC, Wolf SL, Dixit A (2021). Vagus nerve stimulation paired with rehabilitation for upper limb motor function after ischaemic stroke (VNS-REHAB): a randomised, blinded, pivotal, device trial. Lancet.

[6] Edwardson MA, Lucas TH, Carey JR, Fetz EE (2013). New modalities of brain stimulation for stroke rehabilitation. Exp Brain Res.

[7] Levy RM, Harvey RL, Kissela BM, Winstein CJ, Lutsep HL, Parrish TB (2016). Epidural electrical stimulation for stroke rehabilitation: results of the prospective, multicenter, randomized, single-blinded Everest trial. Neurorehabil Neural Repair.

[8] Ackerley SJ, Stinear CM, Barber PA, Byblow WD (2010). Combining theta burst stimulation with training after subcortical stroke. Stroke.

[9] Pomeroy VM, Cloud G, Tallis RC, Donaldson C, Nayak V, Miller S (2007). Transcranial magnetic stimulation and muscle contraction to enhance stroke recovery: a randomized proof-of-principle and feasibility investigation. Neurorehabil Neural Repair.

[10] Vahdat S, Pendharkar AV, Chiang T, Harvey S, Uchino H, Cao Z, et al. Brain-wide neural dynamics of poststroke recovery induced by optogenetic stimulation. Sci Adv. 2021;*7*(33): eabd9465. 10.1126/sciadv.abd9465 PMC8357234 34380610

[11] Cheng MY, Wang EH, Woodson WJ, Wang S, Sun G, Lee AG (2014). Optogenetic neuronal stimulation promotes functional recovery after stroke. Proc Natl Acad Sci.

[12] Wang YC, Galeffi F, Wang W, Li X, Lu L, Sheng H, Hoffmann U, Turner DA, Yang W (2020). Chemogenetics-mediated acute inhibition of excitatory neuronal activity improves stroke outcome. Exp Neurol.

[13] Lee C, Lavoie A, Liu J, Chen SX, Liu BH (2020). Light up the brain: the application of optogenetics in cell-type specific dissection of mouse brain circuits. Front Neural Circuits.

[14] Sternson SM, Roth BL (2014). Chemogenetic tools to interrogate brain functions. Annu Rev Neurosci.

[15] Gomez JL, Bonaventura J, Lesniak W, Mathews WB, Sysa-Shah P, Rodriguez LA (2017). Chemogenetics revealed: DREADD occupancy and activation via converted clozapine. Science.

[16] Manvich DF, Webster KA, Foster SL, Farrell MS, Ritchie JC, Porter JH (2018). The DREADD agonist clozapine N-oxide (CNO) is reverse-metabolized to clozapine and produces clozapine-like interoceptive stimulus effects in rats and mice. Sci Rep.

[17] Cho J, Ryu S, Lee S, Kim J, Kim HI (2020). Optimizing clozapine for chemogenetic neuromodulation of somatosensory cortex. Sci Rep.

[18] Legge SE, Walters JT (2019). Genetics of clozapine-associated neutropenia: recent advances, challenges and future perspective. Pharmacogenomics.

[19] Kim HS, Kim D, Kim RG, Kim JM, Chung E, Neto PR (2014). A rat model of photothrombotic capsular infarct with a marked motor deficit: a behavioral, histologic, and microPET study. J Cereb Blood Flow Metab.

[20] Song H, Jung W, Lee E, Park JY, Kim MS, Lee MC (2017). Capsular stroke modeling based on somatotopic mapping of motor fibers. J Cereb Blood Flow Metab.

[21] Gharbawie OA, Gonzalez CL, Whishaw IQ (2005). Skilled reaching impairments from the lateral frontal cortex component of middle cerebral artery stroke: a qualitative and quantitative comparison to focal motor cortex lesions in rats. Behav Brain Res.

[22] Klein A, Sacrey LA, Whishaw IQ, Dunnett SB (2012). The use of rodent skilled reaching as a translational model for investigating brain damage and disease. Neurosci Biobehav Rev.

[23] Cox RW (1996). AFNI: software for analysis and visualization of functional magnetic resonance neuroimages. Comput Biomed Res.

[24] Papp EA, Leergaard TB, Calabrese E, Johnson GA, Bjaalie JG (2014). Waxholm Space atlas of the Sprague Dawley rat brain. Neuroimage.

[25] Nam MH, Cho J, Kwon DH, Park JY, Woo J, Lee JM (2020). Excessive astrocytic GABA causes cortical hypometabolism and impedes functional recovery after subcortical stroke. Cell Rep.

[26] Cho J, Kwon DH, Kim RG, Song H, Rosa-Neto P, Lee MC (2016). Remodeling of neuronal circuits after reach training in chronic capsular stroke. Neurorehabil Neural Repair.

[27] Kim RG, Cho J, Ree J, Kim HS, Rosa-Neto P, Kim JM (2016). Sensory-parietal cortical stimulation improves motor recovery in severe capsular infarct. J Cereb Blood Flow Metab.

[28] Shijo K, Katayama Y, Yamashita A, Kobayashi K, Oshima H, Fukaya C (2008). c-Fos expression after chronic electrical stimulation of sensorimotor cortex in rats. Neuromodulation.

[29] Yassin L, Benedetti BL, Jouhanneau JS, Wen JA, Poulet JF, Barth AL (2010). An embedded subnetwork of highly active neurons in the neocortex. Neuron.

[30] Lu H, Gallinaro JV, Normann C, Rotter S, Yalcin I. Time course of homeostatic structural plasticity in response to optogenetic stimulation in mouse anterior cingulate cortex. Cereb Cortex. 2022;32(8):1574–92. 10.1093/cercor/bhab281 34607362

[31] Hogan MK, Hamilton GF, Horner PJ (2020). Neural stimulation and molecular mechanisms of plasticity and regeneration: a review. Front Cell Neurosci.

[32] Dimyan MA, Cohen LG (2011). Neuroplasticity in the context of motor rehabilitation after stroke. Nat Rev Neurol.

[33] Small SL, Buccino G, Solodkin A (2013). Brain repair after stroke–a novel neurological model. Nat Rev Neurol.

[34] Colella P, Ronzitti G, Mingozzi F (2018). Emerging issues in AAV-mediated in vivo gene therapy. Mol Ther Methods Clin Dev.

[35] Galvan A, Raper J, Hu X, Pare JF, Bonaventura J, Richie CT (2019). Ultrastructural localization of DREADDs in monkeys. Eur J Neurosci.

[36] Cho J, Hayes RD, Jewell A, Kadra G, Shetty H, MacCabe JH (2019). Clozapine and all-cause mortality in treatment-resistant schizophrenia: a historical cohort study. Acta Psychiatr Scand.

[37] Hu KH, Li YA, Jia W, Wu GY, Sun L, Wang SR (2019). Chemogenetic activation of glutamatergic neurons in the motor cortex promotes functional recovery after ischemic stroke in rats. Behav Brain Res.

[38] Motaharinia M, Gerrow K, Boghozian R, White E, Choi SE, Delaney KR (2021). Longitudinal functional imaging of VIP interneurons reveals sup-population specific effects of stroke that are rescued with chemogenetic therapy. Nat Commun.

[39] Lee MC, Kim RG, Lee T, Kim JH, Lee KH, Choi YD (2020). Ultrastructural dendritic changes underlying diaschisis after capsular infarct. J Neuropathol Exp Neurol.

[40] Cochran SM, McKerchar CE, Morris BJ, Pratt JA (2002). Induction of differential patterns of local cerebral glucose metabolism and immediate-early genes by acute clozapine and haloperidol. Neuropharmacology.

[41] Tsai SJ, Huang YH, Huang HJ, Sim CB (2001). Reduced regional [14C]2-deoxyglucose uptake in response to long-term clozapine administration in rats. Neuropsychobiology.

[42] Cramer SC (2018). Treatments to Promote Neural Repair after Stroke. J Stroke.

[43] Ovbiagele B, Goldstein LB, Higashida RT, Howard VJ, Johnston SC, Khavjou OA (2013). Forecasting the future of stroke in the United States: a policy statement from the American Heart Association and American Stroke Association. Stroke.

[44] Ballester BR, Maier M, Duff A, Cameirao M, Bermudez S, Duarte E (2019). A critical time window for recovery extends beyond one-year post-stroke. J Neurophysiol.

[45] Jones TA, Schallert T (1992). Overgrowth and pruning of dendrites in adult rats recovering from neocortical damage. Brain Res.

[46] Kaur H, Prakash A, Medhi B (2013). Drug therapy in stroke: from preclinical to clinical studies. Pharmacology.

[47] Stroke Therapy Academic Industry R. Recommendations for standards regarding preclinical neuroprotective and restorative drug development. Stroke. 1999;30:2752–8. 10.1161/01.str.30.12.2752 10583007

