# Supplementary material for: Clozapine-Induced Chemogenetic Neuromodulation Rescues Post-Stroke Deficits After Chronic Capsular Infarct
Source: Transl Stroke Res. 2022 Jul 9;14(4):499–512. doi: 10.1007/s12975-022-01059-8 (PMC10300150; doi:10.1007/s12975-022-01059-8)
Supplement: Supplementary file 1 — Supplementary file1 (DOCX 1316 KB) [file 12975_2022_1059_MOESM1_ESM.docx]

**Supplementary Materials for**

**Clozapine-induced Chemogenetic Neuromodulation Rescues Post-Stroke Deficits after Chronic Capsular Infarct**

Jongwook Cho^1^, Seungjun Ryu^1^, Sunwoo Lee^1^, Junsoo Kim^1^, Ji-Young Park^1^, Hyuk-Sang Kwon^1^*, Hyoung-Ihl Kim^1,2^*

^1^Department of Biomedical Science and Engineering, Gwangju Institute of Science and Technology (GIST), Gwangju 61005, Republic of Korea

^2^Department of Neurosurgery, Presbyterian Medical Center, Jeonju 54987, Republic of Korea.

^*^Correspondence to:

Hyuk-Sang Kwon ([hyuksang@gist.ac.kr](mailto:hyuksang@gist.ac.kr)) or Hyoung-Ihl Kim ([hyoungihl@gmail.com](mailto:hyoungihl@gmail.com))

**This file includes:**

Fig. S1. Longitudinal changes in regional glucose metabolism after chemogenetic stimulation

Fig. S2. CLZ-ChemoNM increases BDNF expression in DREADD-expressing neurons.


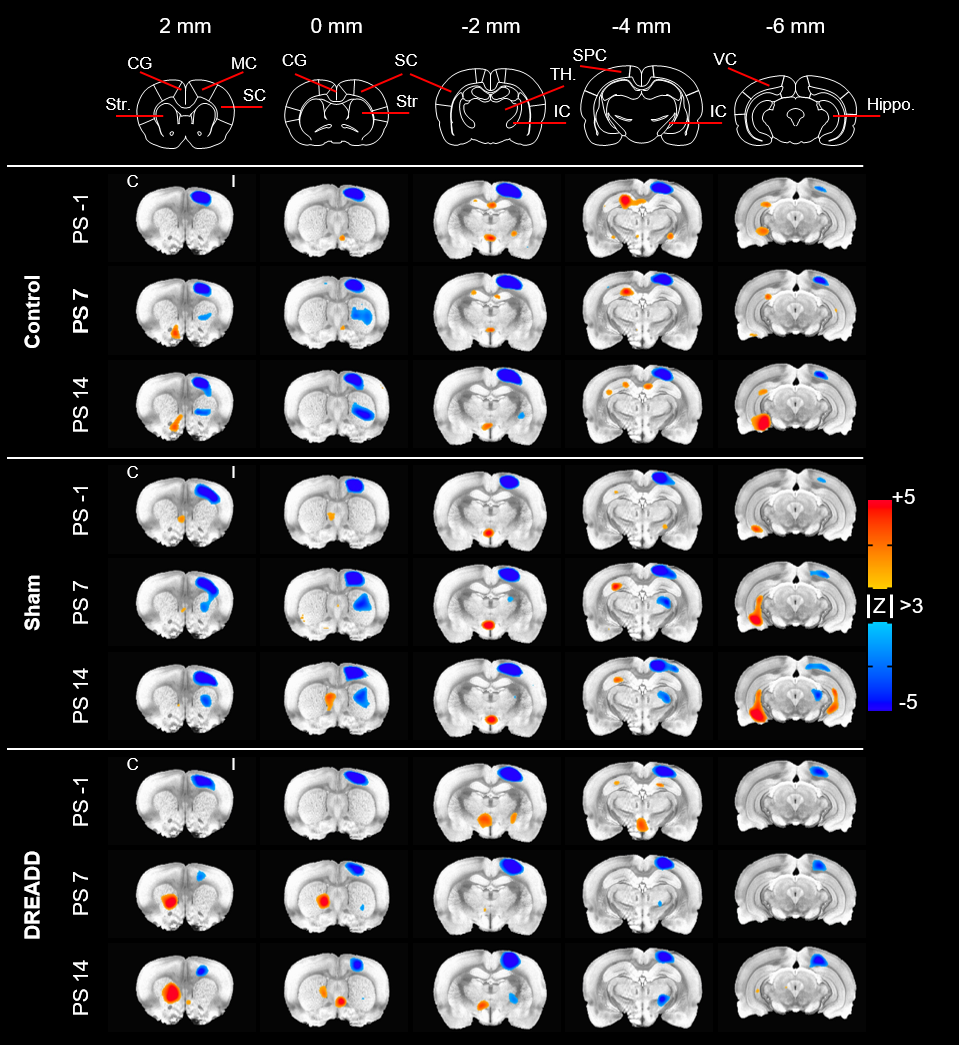


**Fig. S1. Longitudinal changes in regional glucose metabolism after chemogenetic stimulation.** Color-coded maps show the activated and deactivated regions in each group (*N* = 8, 8, and 9 for control, sham, and DREADD groups, respectively).  Image analysis comparing the pre-lesional (base) and post-lesional (PS 0, PS 7 and PS 14) images was performed to assess time-dependent changes in cortical diaschisis and regional glucose metabolism ( 3dLME in AFNI *p* <0.001, false discovery rate q < 0.05). CG; cingulate gyrus, ST; striatum, MC; motor cortex, SC; sensory cortex, IC; internal capsule, TH; thalamus, SPC; sensori-parietal cortex Hippo; hippocampus, C; contralesional, I; ipsilesional.


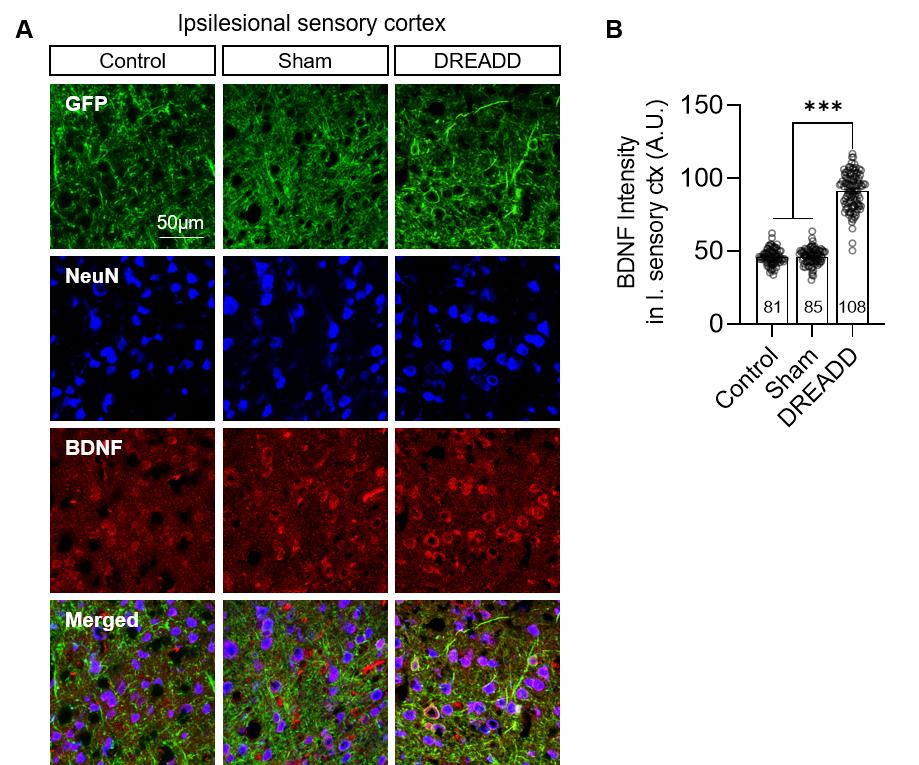


**Fig. S2. CLZ-ChemoNM increases BDNF expression in DREADD-expressing neurons.** (**A**) Representative confocal images of NeuN and BDNF in ipsilesional sensory cortex (N = 4, 4, and 5 for control, sham and DREADD groups, respectively). (**B**) Quantification of BDNF intensity (one image from one animal, *N* = 4, 4, and 5 for control, sham, and DREADD groups, respectively). Animals in DREADD group exhibit significantly higher BDNF expression. (one-way ANOVA with Tukey test, *F*_(2,271)_ = 41.07, *p* < 0.0001). The number on each bar refers to the number of cells analyzed. Data are represented as the mean $\pm$ S.E.M. ****p* < 0.001
